# Synthesis and optimization of biobased carbon adsorbent monoliths from chitosan-polybenzoxazine for efficient CO_2_ capture

**DOI:** 10.1039/d5ra00110b

**Published:** 2025-03-03

**Authors:** José E. Mosquera, Liana Delevingne, Frédéric Delbecq, Elias Daouk, Audrey Drelich, Khashayar Saleh, Rémi Gautier, Mikel Leturia

**Affiliations:** a Université de Technologie de Compiègne, ESCOM, TIMR Compiègne France mikel.leturia@utc.fr +33 6 28 23 87 85; b IMT Nord Europe, Institut Mines-Télécom, CERI Energie et Environnement F-59508 Douai France

## Abstract

The present study introduces a novel method for the preparation of a CO_2_ carbon adsorbent derived from biobased precursors. Porous carbon adsorbents were synthesized through the carbonization and thermal activation of biobased chitosan-polybenzoxazine. First, the study explored the influence of varying amounts of the key polymer precursors, lysine (0.05–0.1 g) and chitosan (0.6–0.12 g), on the surface and adsorption characteristics of the obtained carbons. This aimed to identify the most favourable amounts of these precursors that resulted in the highest CO_2_ adsorption performance. In the subsequent stage, the study investigated the impact of different activation times (1–7 h) to enhance the surface characteristics and CO_2_ adsorption capacity of the activated carbon. Both carbonization and activation processes were conducted in a tubular furnace at 900 °C under N_2_ and CO_2_ atmospheres, respectively. After carbonization, the resulting carbon monoliths exhibited a char yield of approximately 49 wt%, with a BET surface area of up to 541 m^2^ g^−1^ and a CO_2_ uptake of 4.0 mmol g^−1^ at 0 °C and 1 bar. After activation, the obtained samples displayed a surface area in the range of 650–1000 m^2^ g^−1^, with CO_2_ adsorption capacities at 1 bar ranging from 4.5 to 5.6 mmol g^−1^ at 0 °C and 3.2 to 4 mmol g^−1^ at 25 °C. The activated carbons also demonstrated excellent selectivities for CO_2_/N_2_ and CO_2_/CH_4_ mixtures, along with a stable CO_2_ adsorption–desorption performance after 10 cycles.

## Abstract 2

Biobased porous carbon monoliths were synthesized from chitosan-polybenzoxazine. The precursor ratio and activation time were optimized to obtain high CO_2_ uptake and selectivity.

## Graphical abstract

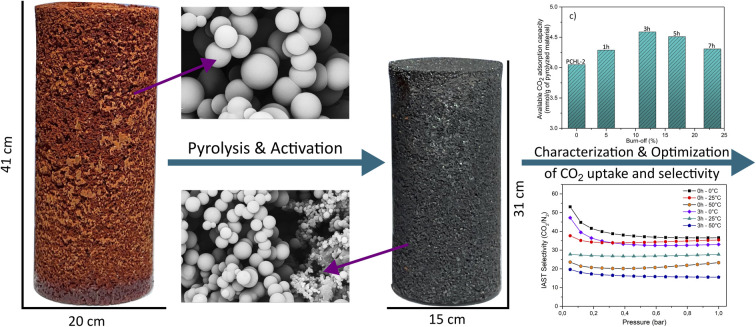

## 1. Introduction

Over the past few decades, global warming has emerged as a critical concern due to increasing levels of greenhouse gases in the atmosphere, primarily caused by the use of fossil carbon resources in energy, industry and transportation.^1^ In particular, carbon dioxide (CO_2_) is widely regarded as the foremost contributor to the global rise in mean temperatures.^2^ As a result, numerous researchers have focused their efforts on developing efficient methods for CO_2_ capture and storage. These efforts aim not only to reduce CO_2_ emissions but also to create strategies for enhancing the selective separation of CO_2_ from complex gas mixtures. Recently, various CO_2_ capture technologies have been under investigation, including chemical absorption, adsorption and membrane separation.^3–6^

Among these technologies, CO_2_ adsorption by solid porous materials has received significant attention, owing to its numerous advantages, including chemical stability, ease of recovery and high adsorption capacity, even under humid conditions.^7–9^ Recent studies have further demonstrated that carbon adsorbents exhibit excellent CO_2_ adsorption capacity and selectivity.^10–12^ Porous carbon materials can be produced through the pyrolysis of various polymeric precursors including polymers of natural origin, as well as synthetic polymers such as polyamide, polyacrylonitrile, phenolic resin and polymer blends.^8,13^ Despite the development of numerous carbonized materials, there is a strong trend toward creating new adsorbent materials from bioresource-derived precursors with superior adsorption capacity. This trend continues to open up opportunities for further advancements in this type of material.

Renewable resources and bio-based raw materials have been successfully utilized as precursors for producing carbon adsorbents, including wood,^14^ chitosan,^15^ polysaccharides^16^ and lignin,^17^ among others, showcasing promising results for CO_2_ capture. Additionally, N-doped carbon frameworks produced from several biomass-derived precursors have shown enhanced CO_2_ adsorption performance under ambient conditions.^18–20^

Polybenzoxazines (PBZ) are a class of phenolic thermosetting resins typically synthesized through a cationic ring-opening polymerization of benzoxazine monomers, forming a crosslinked network of tertiary amine bridges. They can be synthesized from cost-effective raw materials, including primary amines, phenol sources and formaldehyde.^8,11,20–24^ These materials are distinguished from traditional polymers by their exceptional features, such as good chemical and electrical resistance, high thermal stability, good mechanical strength, high char yields, low water adsorption, minimal shrinkage during curing and flame retardancy.^18,25–29^ These unique qualities extend the utility of PBZ across various applications, from serving as adsorbents for CO_2_,^21,30^ water treatment^31^ and also electronics and aerospace industries.^16,32^ In recent times, there has been a growing interest in polybenzoxazines derived from renewable bioresource materials.^25^ Notably, benzoxazine molecules have been synthesized using green solvents, such as water and aqueous solutions.^26^ In this work, we will explore the development of chitosan-based polybenzoxazine-derived carbon materials for CO_2_ capture. For comparison, Table 1 summarizes the CO_2_ adsorption capacity of various polybenzoxazine-derived carbon materials reported in the literature.

**Table 1:** Comparison of the CO_2_ adsorption capacity of different PBZ-based porous carbon materials from the literature

Author	*S*_BET_ [m^2^ g^−1^]	CO_2_ uptake at 0 °C and 1 bar [mmol g^−1^]
Present work	998	5.60
Konnola^23^	910	4.25
Jin^30^	1720	6.96
Zhang^33^	815	7.00
Guo^34^	1292	7.04
Hao^20^	1392	4.90
Hong^22^	2423	8.44
Samy^18^	560	6.81
Xia^35^	3360	6.92

Chitosan (2-amino-2-deoxy-d-glucopyranose) is a natural polymer, primarily derived from the exoskeletons of crab and shrimp shells.^28^ It is soluble in acidic solvents having a pH value lower pH 6.0.^36^ This biopolymer has attracted enormous interest due to its advantageous characteristics, which include reactive functionality, a high concentration of amine groups, low production costs, widespread availability, low toxicity and environmental friendliness.^28,37^ Consequently, chitosan finds applications in various domains, ranging from drug delivery systems,^38^ separation membrane^39^ to active food packaging.^40^ Several carbon adsorbents derived from chitosan-based precursors for CO_2_ capture have been reported. For instance, Witoon *et al.*^41^ synthesized a meso–macroporous polyethyleneimine-loaded silica monolith using chitosan as a biotemplate. The resulting carbon product exhibited a BET surface area of 246 m^2^ g^−1^ and a CO_2_ adsorption capacity of 3.8 mmol g^−1^ at 80 °C. Alhwaige *et al.*^42^ fabricated chitosan-graphene oxide hybrid aerogels through a freeze-drying method. The resulting carbon possessed a BET surface area of 415 m^2^ g^−1^ and a CO_2_ adsorption capacity of 4.15 mmol g^−1^ at 25 °C and 1 bar pressure. In a subsequent study, the authors prepared a carbon aerogel from clay-reinforced biobased chitosan-polybenzoxazine using the same method, yielding a carbon material with high surface areas (up to 710 m^2^ g^−1^) and excellent adsorption performance of 5.72 mmol g^−1^ at 25 °C and 1 bar.^19^ Kamran *et al.*^43^ developed chitosan-based porous carbons through hydrothermal carbonization and chemical activation with KOH and NaOH. The products displayed a superior surface area of 4168 m^2^ g^−1^ and a maximum CO_2_ uptake of 8.36 mmol g^−1^ at 0 °C and 1 bar. Ghimbeu and Luchnikov^15^ produced nitrogen-doped carbonized beads from chitosan acetate, cross-linkers and Pluronic F127 co-solution drops. After freeze-drying and carbonization, the resulting carbon achieved a surface area of 433 m^2^ g^−1^ and a CO_2_ uptake of 2.85 mmol g^−1^ at 0 °C at 1 bar.

Within the preceding scope, the present study reports a simple, scalable and eco-friendly approach for preparing carbon adsorbent monoliths for CO_2_ adsorption. The formation of carbon monoliths is achieved through the utilization of biobased precursors, wherein chitosan serves as both a bio-templating and nitrogen source, lysine functions as a polymerization agent, and water serves as the exclusive solvent. Subsequently, the resulting polymer monolith undergoes drying at 75 °C, followed by carbonization and thermal activation. Therefore, the aim of this work is to fabricate a porous carbon monolith derived from chitosan-polybenzoxazine, and enhance its properties by thermal activation, including surface characteristics and adsorption capacity. The production yields, CO_2_/N_2_ and CO_2_/CH_4_ selectivities, as well as adsorption/desorption cyclability are also evaluated. It is noteworthy that this study highlights a method with the following key advantages: it mainly uses sustainable biobased precursors, still offering simplicity and scalability, and includes optimized formulation and fabrication processes, making it well suited for industrial applications.

## 2. Materials and methods

### 2.1. Materials

Chitosan (≥75% deacetylated from shrimp), resorcinol (99.0%) and formalin (37 wt% in water), were purchased from Sigma-Aldrich Corp. Lysine (98%) was purchased from Acros Organics, and APG (alkyl polyglycoside C_8_–C_10_, trade name Plantacare® 2000 UP) from BASF. All chemicals were used as received.

### 2.2. Preparation of chitosan-PBZ carbon monolith

**Fig. 1:**
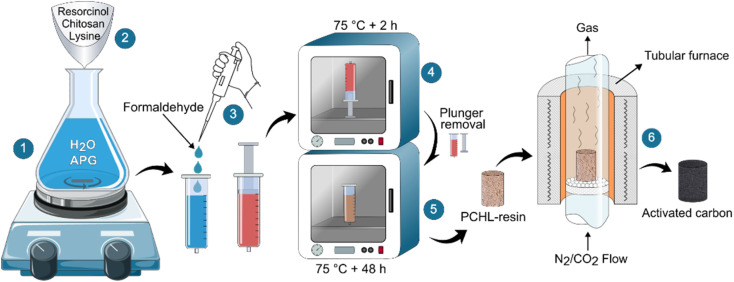
Schematic of the experimental procedure for activated carbon preparation.

The chitosan-PBZ monolith was synthesized *via* a sol–gel process, as shown in Fig. 1. In a typical synthesis process, 0.2 g of APG was dissolved in 13 mL of deionized water, in a 50 mL flask with magnetic stirring under ambient conditions (step 1). Afterward, resorcinol (27.3 mmol, 3 g), lysine (0.1 g) and chitosan (0.9 g) were added to this solution. The mixture was vigorously stirred until complete dissolution of resorcinol and lysine, leaving a uniform suspension of chitosan (step 2). The resulting suspension was poured into a 25 mL plastic syringe and subsequently, formaldehyde (54.5 mmol, 1.63 g, corresponding to 4 mL of formalin at 37 wt% in water) was quickly added to the solution (step 3). Then, the syringe containing the suspension was vigorously agitated and placed vertically in an oven, preheated to 75 °C for 2 hours (step 4). After that, the remaining liquid and the syringe plunger were removed, and the syringe barrel was returned to the oven at the same temperature, for an additional 48 hours to achieve complete drying (step 5).

The as-prepared polymer monolith was extracted from the syringe and pyrolyzed for 2 hours in a tubular furnace at 900 °C (with a heating rate of 5 °C min^−1^), under nitrogen atmosphere (with a gas flow rate of 200 mL min^−1^) (step 6). In this work, the selection of the pyrolysis temperature (900 °C), heating rate (5 °C min^−1^) and hold time (2 hours) was based on a comprehensive review of existing literature, preliminary experiments, and insights derived from prior research.^44^

As a first study, three polymer monoliths were prepared, each with different amounts of chitosan and lysine (corresponding to low, medium and high concentrations, as detailed in Table 2), while the mass of the remaining precursors stayed constant. These polymer monoliths were denoted as PCHL-*x* (polybenzoxazine–chitosan–lysine), where “*x*” indicates the concentration level of chitosan and lysine in the resulting material (*x* = 1 for low concentration, *x* = 2 for medium concentration and *x* = 3 for high concentration). Subsequently, the three polymer monoliths were pyrolyzed and the CO_2_ adsorption capacity of the obtained porous carbons was characterized (characterization techniques are detailed in section 2.3).

**Table 2:** Concentration levels of chitosan and lysine in the polymer monoliths

Sample name	Concentration level	Chitosan [g]	Lysine [g]
PCHL-1	Low	0.6	0.05
PCHL-2	Medium	0.9	0.1
PCHL-3	High	1.2	0.15

As a second study, the pyrolyzed sample displaying the highest CO_2_ adsorption capacity was chosen for further investigation regarding the subsequent thermal activation step. In order to optimize the surface characteristics and adsorption capacity, different activation times (1, 3, 5 and 7 hours) were applied to the pyrolyzed monolith previously selected. In all these experiments, the carbon monolith was heated from room temperature to 900 °C, with a ramp rate of 20 °C min^−1^ under nitrogen atmosphere.

The flowing gas was subsequently switched from nitrogen to a gas mixture of CO_2_/N_2_ (20/80 molar%), maintained for the selected activation time, and then switched back to nitrogen, all at the same flow rate (200 mL min^−1^). The resulting activated carbons were designated as ACP-*x-y* (activated carbon polybenzoxazine), where “*x*” indicates the concentration level of chitosan and lysine in the polymer monolith, and “*y*” refers to the activation time (in hours).

### 2.3. Characterization techniques

Fourier Transform Infrared (FTIR) spectroscopy was used to identify the functional groups of the PBZ materials and the porous carbons, using a Thermo Scientific Nicolet iS5 with iD1 transmission FTIR spectrometer (Thermo 190 Scientific®, USA) at room temperature in the range of 400–4000 cm^−1^ at a resolution of 4 cm^−1^, with a KBr pellet. Elemental analysis was carried out on a Thermo Scientific Flash 2000 CHNS/O Analyzer (Thermo Fisher Scientific, USA). The surface topography of porous carbon materials was observed by Scanning Electron Microscopy (SEM), with a FEI Quanta 3D FIB FEG instrument operated at 20 kV. A 3Flex sorption analyser (Micromeritics, Norcross, GA, USA) was used to assess the surface characteristics and gas adsorption properties, by measuring the isotherms for CO_2_, N_2_ and CH_4_. N_2_ adsorption/desorption isotherms were conducted at −196 °C using nitrogen (99.998% purity), after degassing the porous carbons at 220 °C for 20 h. The specific surface area (*S*_BET_) was calculated from the N_2_ adsorption isotherm by using the Brunauer–Emmett–Teller (BET) method, while the pore size distribution was estimated with the Horvath–Kawazoe (HK) method. The total pore volume (*V*_total_) was calculated from the amount of N_2_ adsorbed at a relative pressure of 0.99. Micropore volume (*V*_mic_) and micropore surface area (*S*_mic_) were calculated using the *t*-plot method. The adsorption isotherms of CO_2_, N_2_ and CH_4_ were conducted at three different temperatures (0, 25 and 50 °C) in the pressure range of 0 to 1 bar. Prior to each adsorption test, the samples were degassed at 220 °C for at least 6 hours.

The char yield refers to the amount of carbon obtained after the carbonization process, expressed as a percentage of the initial mass of the precursor material. It was calculated based on the following equation:1
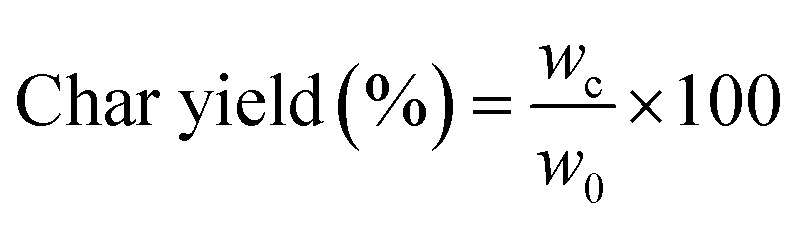
where *w*_0_ (g) and *w*_c_ (g) are the initial mass of the precursor and mass of the carbon material after pyrolysis, respectively.

The burn-off reflects the activation progress and is calculated as the percentage of mass lost during the activation of the carbonized material:2
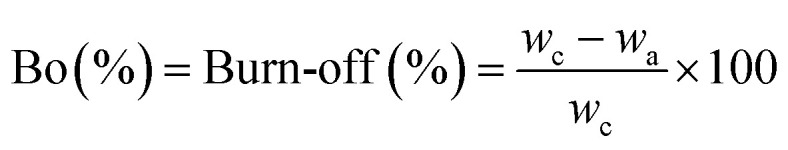
where *w*_a_ (g) is the final mass of activated material (*i.e.*, mass after activation).

The activation process leads to a higher surface area but also results in a mass loss of the activated product (burn-off). Thus, determining the optimal balance between CO_2_ adsorption capacity and available mass of activated carbon is crucial. To address this, we introduce the concept of “available CO_2_ adsorption capacity”, expressed in mmol of CO_2_ per gram of pyrolyzed material. This requires converting the CO_2_ adsorbed per unit mass of activated material (denoted as *A*_c_) into CO_2_ adsorbed per unit mass of pyrolyzed material. Consequently, the available CO_2_ adsorption capacity is calculated as:3
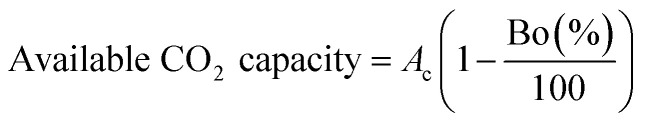
where *A*_c_ (mmol of CO_2_ g^−1^ of activated material) is the CO_2_ adsorption capacity of the activated material and Bo is the burn-off (%) after activation.

The CO_2_/N_2_ and CO_2_/CH_4_ selectivities of the carbon adsorbent were calculated using the Ideal Adsorbed Solution Theory (IAST),^5,22^ given by:4
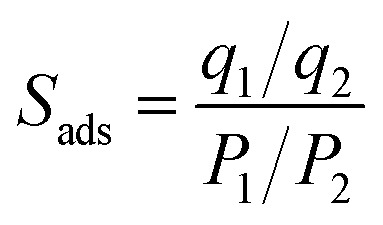
where *q*_i_ is the amount of gas i adsorbed (mmol g^−1^) and *P*_i_ is the partial pressure (bar) of gas i in the mixture.

## 3. Results and discussion

### 3.1. Structural properties of the polymer and porous carbon monoliths

**Fig. 2:**
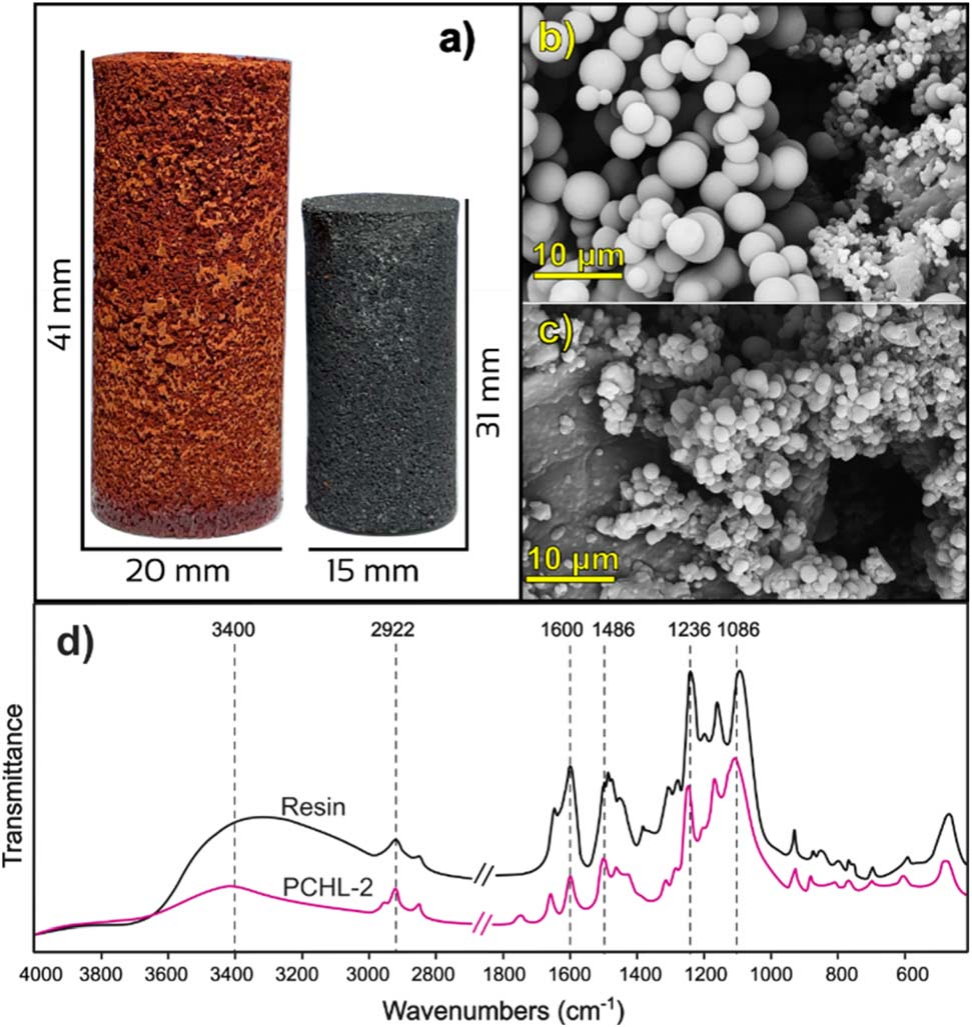
(a) Representative photographs of polymer and carbon monoliths; (b) and (c) SEM images of resin and carbonized materials, respectively; (d) FTIR spectra of resin and carbonized materials.

Porous carbon monoliths were successfully prepared from chitosan-PBZ polymers. In Fig. 2a, a representative example of chitosan-PBZ polymer monolith and its carbonized counterpart are presented. Notably, the carbonization process yields a crack-free carbon monolith that retains a uniform shape, similar to the original polymer structure. The resulting carbon monolith typically exhibits dimensions of 31 mm in length and 15 mm in diameter, which corresponds to an overall volume shrinkage of 60%. SEM images (Fig. 2b and c) further illustrate that both the polymer and carbon materials consist of interconnected microspheres and possibly amorphous solid, forming a microporous framework. The FTIR spectra of both the precursor polymer and the porous carbon are presented in Fig. 2d. The FTIR spectrum of chitosan-PBZ resin exhibits characteristic absorption peaks in the range of 3400–3200 cm^−1^ attributed to the O–H bonds and/or N–H stretching vibrations of chitosan amide groups.^39^ Additionally, bands at 2922 cm^−1^ and 2847 cm^−1^ correspond to C–H bending. The absorption peaks in the range of 1600 cm^−1^ and 1485 cm^−1^ can be attributed to C=C and C–C within the aromatic rings of the benzoxazine structure.^30,45^ Furthermore, the bands observed at 1240 and 1160 cm^−1^ were assigned to asymmetric and symmetric stretching of the oxazine structure C–O–C.^27^ Moreover, a band around 1110 cm^−1^ suggests the presence of the C–N stretching vibrations,^46^ while the weak absorption bands occurring at 926, 874 and 764 cm^−1^ are indicative of C–H groups.

### 3.2. Effect of chitosan and lysine concentrations

**Fig. 3:**
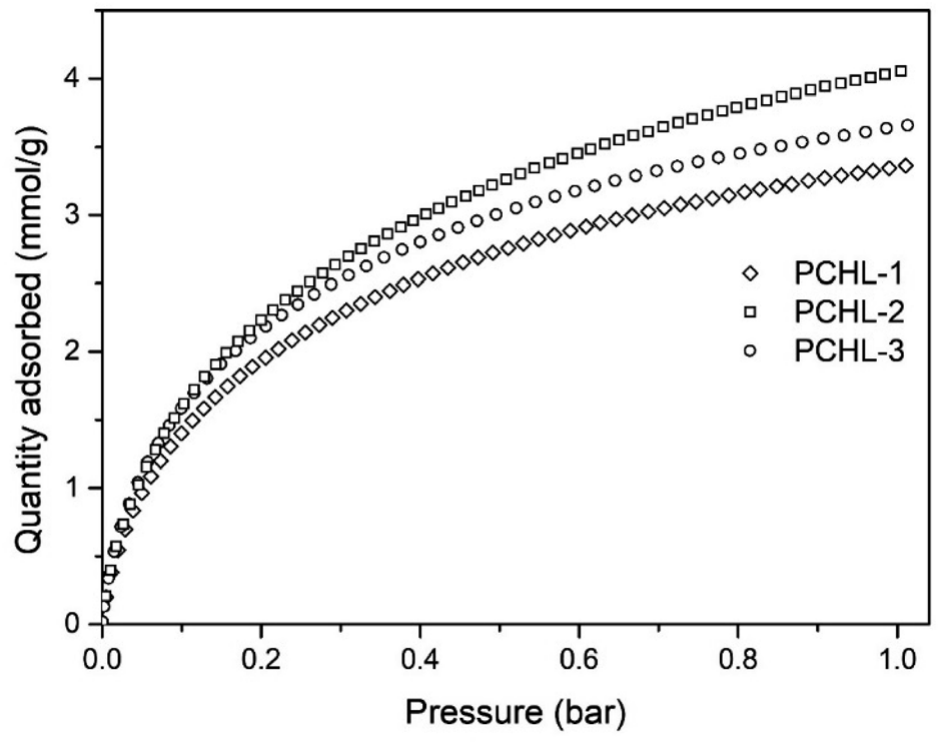
CO_2_ adsorption isotherms at 0 °C of chitosan-based porous carbon monoliths.

As noted earlier, three monoliths with varying amounts of lysine and chitosan were synthesized and carbonized (while the mass of the remaining precursors stayed constant). The CO_2_ adsorption isotherms, measured at 0 °C, are illustrated in Fig. 3. The highest CO_2_ uptake capacity at 0 °C was achieved with the PCHL-2 sample. To understand this behaviour, Table 3 presents the key characteristics of the three monoliths. The data reveals an almost constant char yield for all the samples of approximately 49 wt%. In addition, elemental analysis demonstrated an increase in nitrogen content with the addition of chitosan and lysine, ranging from 1.7 to 2.3 wt%. This observation aligns with expectations, considering that both precursors can be considered as nitrogen sources. Furthermore, the results (Table 3 and Fig. 3) reveal that the highest BET surface area (541 m^2^ g^−1^) and CO_2_ uptake capacity at 0 °C and 1 bar (4.05 mmol g^−1^) were achieved with the PCHL-2 sample. These results suggest that the cross-linking of chitosan and lysine at an intermediate concentration level results in an optimized carbon framework structure, with increased surface area and active sites for CO_2_ adsorption.

**Table 3:** Adsorption characteristics of chitosan-based porous carbon monoliths

Sample	Chitosan and lysine concentration level	Char yield [%]	*S*_BET_ [m^2^ g^−1^]	CO_2_ uptake at 0 °C and 1 bar [mmol g^−1^]	Elemental analysis (wt%)		
					N	C	H
PCHL-1	Low	51%	489	3.36	1.73	93.41	0.40
PCHL-2	Medium	49%	541	4.05	2.07	91.47	0.37
PCHL-3	High	47%	506	3.66	2.30	93.70	0.41

According to the work of Sun *et al.*,^47^ a structured network made of chitosan and other elements, such as amino acids, could generate nitrogen-enriched substructures, including pyridinic or graphitic moieties, after pyrolysis. These substructures are reported to be important for the stabilization of the CO_2_ uptake. Consequently, the PCHL-2 formula was selected as the preferred material for the subsequent thermal activation experiments.

### 3.3. Effect of activation progress

The carbon material obtained after pyrolysis of the chitosan-PBZ polymer was subjected to activation with CO_2_ at 900 °C, in order to enhance its surface characteristics and CO_2_ adsorption capacity. The evolution of the textural properties (specific surface area, micropore surface area, total and micropore volumes) with activation progress (*i.e.*, burn-off) is summarized in Table 4, together with the CO_2_ adsorption capacity and elemental analysis.

**Table 4:** Textural and adsorption properties of porous carbons at different stages of activation

Sample	Burn-off [%]	*S*_BET_ a [m^2^ g^−1^]	*S*_mic_ b [m^2^ g^−1^]	*V*_total_ c [cm^3^ g^−1^]	*V*_mic_ d [cm^3^ g^−1^]	CO_2_ uptake [mmol g^−1^]		Elemental analysis [wt%]			
						0 °C	25 °C	N	C	H	O
PCHL-2	0	541	501	0.28	0.25	4.05	3.25	2.07	91.47	0.37	1.53
ACP-2-1	5	660	622	0.33	0.32	4.52		2.16	91.74	0.53	1.22
ACP-2-3	12	930	845	0.46	0.42	5.22	3.99	1.98	92.60	0.49	1.11
ACP-2-5	17	995	888	0.49	0.45	5.44		1.87	92.48	0.42	1.02
ACP-2-7	23	998	902	0.50	0.46	5.60	3.95	1.84	92.32	0.42	1.29

a BET specific surface area obtained from the adsorption data in the *P*/*P*_0_ range from 0.05 to 0.2.

b Microporous specific surface area obtained from *t*-plot method.

c Total pore volume at a relative pressure of 0.99.

d Micropore volume. The results are shown as mean values (*n* = 2 replicates).

First, it is evident that the burn-off increases with activation time, ranging from 5% to 23% within the studied time range (1 to 7 hours). This increase is expected due to the extended contact time between CO_2_ and the carbon material during the activation treatment. However, the resulting activated carbon monoliths did not show any significant modifications in their physical structure during the activation process, as the shrinkage did not exceed 5% under all studied conditions. In the same way, there is no clear trend observed in the elemental composition, particularly in terms of nitrogen content. The samples still contain approximately 2 wt% of nitrogen after activation. These nitrogen content values are of interest due to the potential role that N heteroatoms can play in CO_2_ capture.^8,48^

**Fig. 4:**
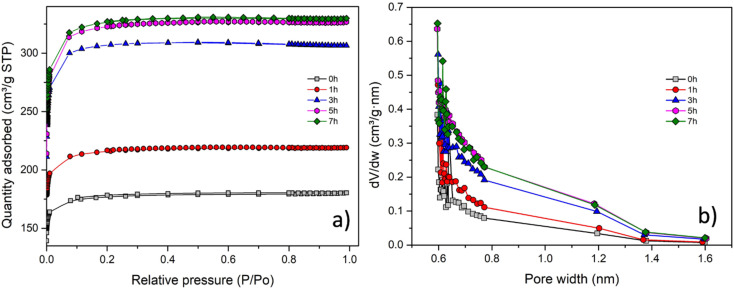
(a) N_2_ sorption isotherms for different activation times. (b) Pore size distributions (PSDs) calculated from the Horvath–Kawazoe method.

The nitrogen adsorption/desorption isotherms and the corresponding pore-size distributions of the obtained carbon materials are displayed in Fig. 4a and b, respectively. As shown in Fig. 4a, the porous carbon materials exhibit typical type-I isotherms, with a rapid increase of N_2_ sorption at low relative pressures (<0.05), indicating the predominance of micropores for all of these samples.

From Table 4, a progressive increase of the main surface characteristics (specific surface area, micropore surface area, total and micropore volumes) can be observed with the activation progress. For instance, *S*_BET_ increased from 541 to 998 m^2^ g^−1^, while *V*_total_ increased from 0.28 to 0.50 cm^3^ g^−1^. This trend is consistent with findings in prior studies on CO_2_ activation of carbons.^49–51^ The enhancement of these textural properties can be attributed to the prolonged contact time between CO_2_ and the char, leading to increased burn-off and greater pore development.^52^ Also, it should be noted that the increase of the main surface characteristics becomes slower in the later stages of activation. The Horvath–Kawazoe micropore size distributions of the obtained porous carbons are presented in Fig. 4b. The majority of the micropores are concentrated in the 0.6–1.2 nm range, with the cumulative pore volume increasing as activation progresses. It can be concluded that thermal activation significantly improves the surface properties of the carbon material. Notably, through this process, the activated carbon achieves up to twice the specific surface area *S*_BET_ and pore volume *V*_total_, compared to the non-activated carbon.

**Fig. 5:**
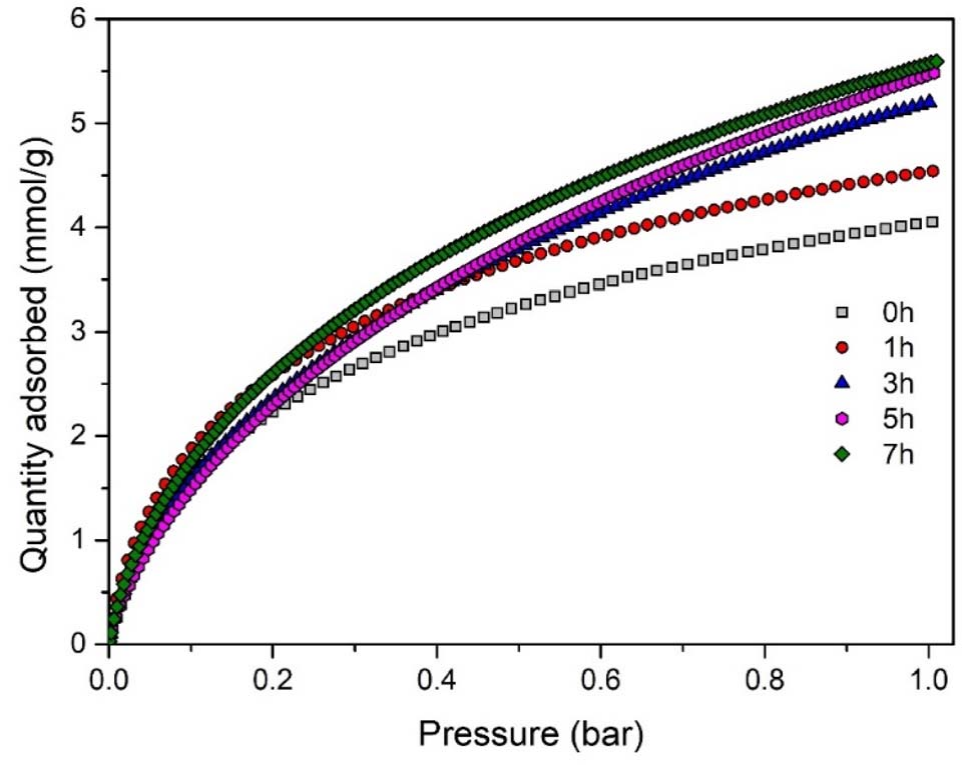
CO_2_ adsorption isotherms at 0 °C for different activation times.

Finally, the CO_2_ adsorption capacity of the porous carbon materials was investigated at 0 °C under atmospheric pressure (1 bar). Table 4 provides a summary of the CO_2_ uptakes and Fig. 5 shows the CO_2_ adsorption isotherms at 0 °C. From both Table 4 and Fig. 5, it can be noticed that the CO_2_ adsorption capacity progressively increases with the activation progress.

The highest CO_2_ adsorption capacity of 5.6 mmol g^−1^ is achieved after 7 h of activation, corresponding to a burn-off of 23%. Notably, this represents an increase in adsorption capacity of up to 38% compared to non-activated carbon. This enhancement is consistent with the earlier trends observed for the BET surface area and pore volume. Furthermore, as the activation progresses, there is an enlargement of the micropore range, especially in the fraction of micropores smaller than 0.8 nm (Fig. 4b). This factor is likely to contribute to the improvement of CO_2_ uptake, as the CO_2_ adsorption capacity at 1 bar is highly dependent on the micropores in this size range.^5,34,48,50^ It is also noteworthy that the resulting carbon materials exhibit a very high CO_2_ uptake under ambient conditions, ranging from 3.25 to 4 mmol g^−1^ at 25 °C and 1 bar.

### 3.4. Production yields of the carbon adsorbents

**Fig. 6:**
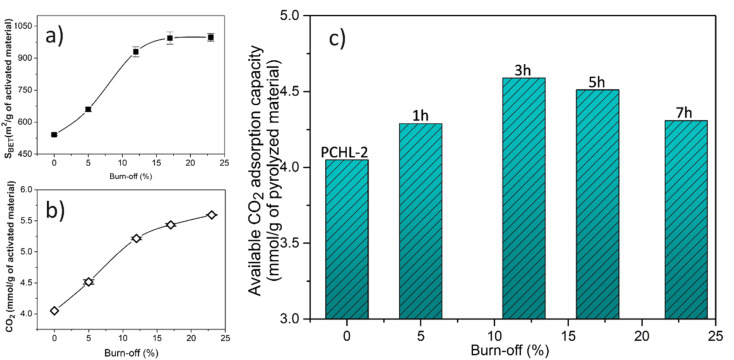
Evolution of the (a) BET surface area, (b) CO_2_ uptake (at 0 °C and 1 bar), and (c) available CO_2_ adsorption capacity of the activated carbons with burn-off.

Fig. 6 shows the evolution of the BET surface area, CO_2_ uptake (at 0 °C and 1 bar) and available CO_2_ adsorption capacity of the activated carbons with burn-off. As explained in the previous section, the activation process leads to an enhancement of textural properties and CO_2_ adsorption capacity (Fig. 6a and b). However, a higher activation progress also leads to a higher mass loss (burn-off), *i.e.*, a lower quantity of the final adsorbent material. Therefore, in the context of industrial applications, it becomes imperative to identify the best compromise between CO_2_ adsorption capacity and available mass of activated carbon. To this purpose, the available CO_2_ adsorption capacity was calculated for all activated carbons, using eqn (3). Fig. 6c displays the CO_2_ adsorption capacity per unit mass of pyrolyzed carbon as a function of the activation burn-off.

The obtained results show that the carbon adsorbent ACP-2-3, obtained after 3 hours of activation (corresponding to a burn-off of 12%), exhibits the best balance between adsorption capacity and mass loss. Accordingly, a recommended route for producing an optimal carbon adsorbent involves carbonizing at 900 °C the chitosan-PBZ monolith with an intermediate concentration of chitosan and lysine, followed by a 3 hour thermal activation process.

### 3.5. CO_2_/N_2_ and CO_2_/CH_4_ selectivities

**Fig. 7:**
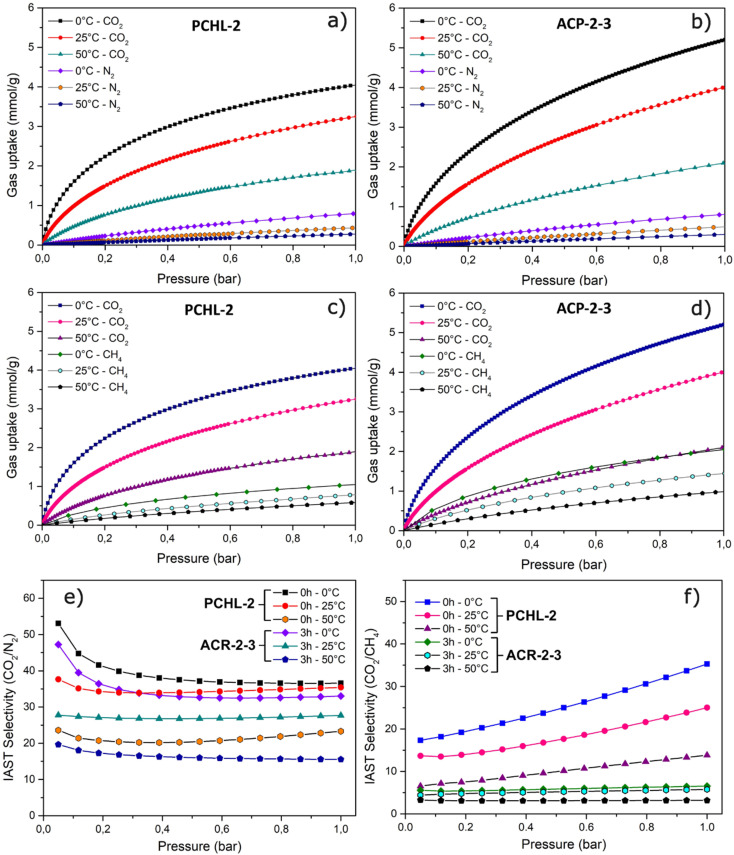
CO_2_ and N_2_ adsorption isotherms for (a) PCHL-2 and (b) ACP-2-3; CO_2_ and CH_4_ adsorption isotherms for (c) PCHL-2 and (d) ACP-2-3; IAST selectivity analyses for (e) (15 : 85) CO_2_/N_2_ mixture and (f) (50 : 50) CO_2_/CH_4_ mixture, for PCHL-2 (*t* = 0 h) and ACP-2-3 (*t* = 3 h).

A crucial aspect in the development of a CO_2_ adsorbent is its ability to separate CO_2_ from other gases, particularly N_2_ (for CO_2_ capture from flue gas streams) and CH_4_ (for natural gas sweetening). Therefore, an effective adsorbent material with high adsorption performance should also exhibit a high CO_2_ selectivity over N_2_ and/or CH_4_. As a consequence, the CO_2_/N_2_ and CO_2_/CH_4_ selectivities of the non-activated porous carbon (PCHL-2) and the chosen activated porous carbon (ACP-2-3) were investigated. To this end, the N_2_ and CH_4_ adsorption isotherms were characterized at three temperatures (0, 25 and 50 °C) for pressures ranging from 0 to 1 bar. The obtained results are presented in Fig. 7 and summarized in Table 5.

**Table 5:** Gas uptakes (CO_2_, CH_4_, and N_2_) at 1 bar and IAST selectivity (CO_2_/N_2_ and CO_2_/CH_4_) for PCHL-2 and ACP-2-3

Sample	CO_2_ uptake [mmol g^−1^]			N_2_ uptake [mmol g^−1^]			CH_4_ uptake [mmol g^−1^]			IAST selectivity (at 1 bar)					
										CO_2_/N_2_ (15 : 85)			CO_2_/CH_4_ (50 : 50)		
	0 °C	25 °C	50 °C	0 °C	25 °C	50 °C	0 °C	25 °C	50 °C	0 °C	25 °C	50 °C	0 °C	25 °C	50 °C
PCHL-2	4.0	3.2	1.9	0.79	0.43	0.27	1.0	0.8	0.6	36.6	35.4	23,3	35.3	25.0	13.8
ACP-2-3	5.2	4.0	2.1	0.82	0.49	0.30	2.1	1.0	1.0	33.0	28.0	16.0	6.5	5.7	3.2

The N_2_ (Fig. 7a and b) and CH_4_ (Fig. 7c and d) adsorption capacities of PCHL-2 and ACP-2-3 measured at 1 bar pressure and 0 °C, 25 °C and 50 °C, range from 0.27 to 0.82 and 0.6 to 2.1 mmol g^−1^, respectively. On the other hand, the CO_2_ adsorption capacity for the same materials under the same conditions ranges from 1.9 to 5.2 mmol g^−1^. This observation indicates significantly lower N_2_ and CH_4_ uptakes compared to that of CO_2_ under the same conditions, suggesting the preferential adsorption of CO_2_ over N_2_ and CH_4_.

The selectivity (*S*_ads_) performance of the binary mixtures of CO_2_/N_2_ and CO_2_/CH_4_ was estimated using the Ideal Adsorbed Solution Theory (IAST), as defined in section 2.3 (eqn (4)). In the 0–1 bar range, the CO_2_/N_2_ selectivity (Fig. 7e) was calculated considering a typical flue gas composition of 15% CO_2_ and 85% N_2_, whereas for CO_2_/CH_4_ selectivity (Fig. 7f), a 50 : 50 mixture was considered (natural gas sweetening). As shown in Table 5, PCHL-2 demonstrates CO_2_/N_2_ IAST selectivity factors (at 1 bar) of 36, 35 and 23, while ACP-2-3 exhibits factors of 33, 28, and 16 at 0 °C, 25 °C and 50 °C, respectively. These results indicate that the carbon adsorbent is effective in selectively separating CO_2_ from N_2_, particularly at the lowest studied temperature (close to 0 °C), where the material demonstrated the highest selectivity. The same IAST selectivity analysis for the CO_2_/CH_4_ (50 : 50) mixture shows that at 1 bar pressure and for temperatures ranging from 0 °C to 50 °C, PCHL-2 displays IAST selectivity factors between 14 and 36, whereas ACP-2-3 shows lower factors ranging from 3 to 6. Similar to the CO_2_/N_2_ mixture, it is evident that the carbon material shows a higher selectivity for CO_2_/CH_4_ separation at temperatures close to 0 °C.

However, it is noteworthy that the selectivity of the non-activated carbon (PCHL-2) is higher than that of the activated carbon (ACP-2-3), particularly for the separation of a CO_2_/CH_4_ (50 : 50) mixture. In fact, materials with smaller average pore sizes are expected to exhibit higher selectivity for CO_2_ over CH_4_.^53^ CO_2_, which has a higher quadrupole moment than CH_4_, interacts more strongly with carbon surfaces within small pores, particularly ultramicropores (<0.7 nm), leading to preferential adsorption.^54^ As shown in Fig. 4b, activation leads to the formation of wider pores in activated carbons compared to the non-activated carbon. This pore enlargement enhances CH_4_ adsorption, thereby decreasing CO_2_ selectivity. From an industrial perspective, these results suggest the activation step could potentially be omitted from the porous carbon fabrication process. However, other factors must be considered, such as the quantity of adsorbent required, contact time, and additional process parameters that could influence the overall performance and efficiency of an industrial process. Breakthrough curves would be of great interest for process optimization, as they would allow adsorption capacity and selectivity assessment under dynamic flow conditions. From a theoretical standpoint, these results demonstrate that selectivity clearly does not solely depend on the specific surface area but is also likely influenced by other factors such as porous structure and heteroatoms, which undergo alterations during activation and may consequently impact selectivity. Further investigation would be required to clearly identify the mechanisms leading to the decrease in selectivity after activation.

Based on all these results, the adsorbent material has demonstrated high CO_2_ adsorption capacity and good selectivities for CO_2_/N_2_ and CO_2_/CH_4_ separations. Consequently, the porous carbon material developed in the present study is suitable for industrial applications in gas separation, such as natural gas sweetening (CO_2_/CH_4_ separation) or the removal of CO_2_ from flue gas (CO_2_/N_2_ separation).

### 3.6. Adsorption/desorption cyclability

**Fig. 8:**
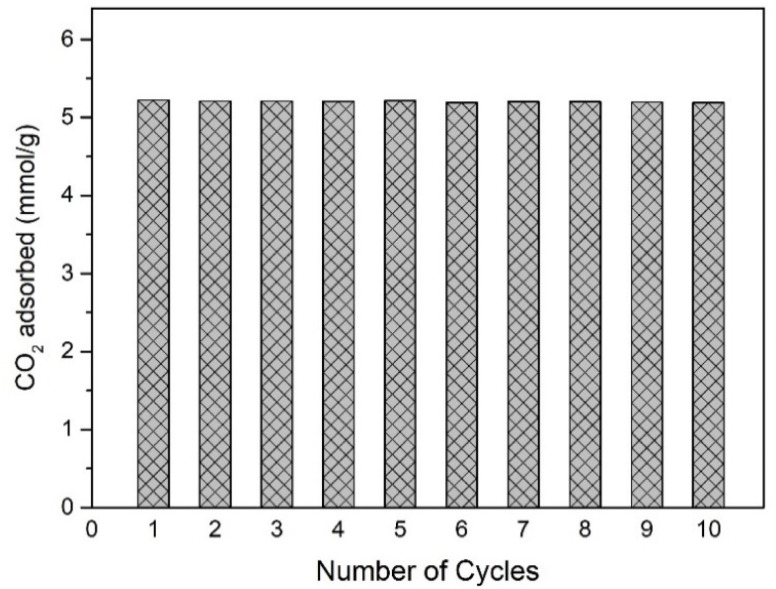
Cyclical CO_2_ adsorption behaviour of the ACP-2-3 activated carbon: adsorption at 0 °C and 1 bar, and desorption at 130 °C under vacuum.

Another crucial aspect to investigate for industrial applications is the stability of the adsorption capacity after multiple adsorption–desorption cycles. In this study, one cycle consisted of a desorption stage at 130 °C under vacuum to desorb CO_2_, followed by an adsorption stage at 0 °C and 1 bar to measure the CO_2_ adsorption capacity at equilibrium. The 3Flex sorption analyser (Micromeritics, Norcross, GA, USA) was used to evaluate the CO_2_ adsorption capacity under equilibrium conditions. This adsorption–desorption cycle was repeated 10 times consecutively on the same activated carbon (ACP-2-3). The results are presented in Fig. 8 and show that even after 10 cycles, the CO_2_ adsorption capacity remains stable. This indicates that ACP-2-3 possesses excellent adsorption–desorption cyclability, which is of great interest for industrial applications.

## 4. Conclusions

This study introduces a straightforward methodology for the fabrication of microporous carbon monoliths from biobased chitosan-PBZ polymers for CO_2_ separation. The quantity of chitosan and lysine was found to influence the textural and adsorption properties of the resulting carbon, with medium concentrations providing the most favourable characteristics. Conversely, the char yield remained nearly constant (∼49 wt%) for the three concentrations studied.

The thermal activation process significantly enhanced the adsorption capacity of the porous carbon monolith. A higher activation progress contributed to greater pore development in the carbonaceous material, resulting in a notable improvement in textural characteristics and adsorption capacity. The activated carbon exhibited a maximum surface area and CO_2_ uptake of around 1000 m^2^ g^−1^ and 5.6 mmol g^−1^, respectively, with a burn-off of 23%.

Analysis of the available CO_2_ adsorption capacity revealed that the activated material achieved the most favourable compromise between increased adsorption capacity and mass loss after a 3 hour activation process (corresponding to a burn-off of 12%). This activated carbon also demonstrated good selectivities for CO_2_/N_2_ and CO_2_/CH_4_ separation, with excellent adsorption–desorption cyclability.

A very important result of this work is that activation may sometimes lead to a decrease in selectivity. This was particularly evident for the separation of the CO_2_/CH_4_ (50 : 50) mixture. From an industrial perspective, this suggests that the activation step could potentially be omitted from the porous carbon fabrication process. Further investigation would be required to clearly identify the mechanisms leading to the decrease in selectivity after activation.

In summary, the advantages of easy preparation, favourable surface and adsorption properties, good selectivity and regeneration capacity, position the obtained adsorbent carbons (non-activated and activated) as promising candidates for industrial CO_2_ separation applications.

## Data availability

The authors declare that the data supporting the findings of this study are available within the paper. Should any raw data files be needed in another format, they are available from the corresponding author upon request. Source data are provided in this paper.

## Author contributions

José E. Mosquera: data curation, formal analysis, investigation, methodology, resources, validation, visualization, writing – original draft. Liana Delevingne: data curation, investigation, validation, visualization. Frédéric Delbecq: conceptualization, formal analysis, methodology, writing – review & editing. Elias Daouk: conceptualization, formal analysis, methodology, writing – review & editing. Audrey Drelich: conceptualization, formal analysis, methodology, writing – review & editing. Khashayar Saleh: conceptualization, project administration, supervision, writing – review & editing. Rémi Gautier: conceptualization, funding acquisition, methodology, project administration, writing – review & editing. Mikel Leturia: conceptualization, funding acquisition, methodology, project administration, resources, supervision, writing – review & editing.

## Conflicts of interest

There are no conflicts to declare.
